# Thermo-mechanical loads of confined sea ice on structures

**DOI:** 10.1098/rsta.2017.0341

**Published:** 2018-08-20

**Authors:** Aleksey Marchenko

**Affiliations:** The University Centre in Svalbard, PO Box 156, 9171 Longyearbyen, Norway

**Keywords:** sea ice, coastal structures, loads, tides, thermal expansion

## Abstract

Records of the ice pressures on the joggle skirt of the coal quay in Spitsbergen were performed over winter seasons in 2013 and 2015. The ice thickness below the quay was above 2 m. Ice temperature over the ice thickness and water pressure at the sea bed were measured synchronously. It was discovered that sea water migrates through the ice confined inside the joggle skirt under the influence of tidal changes of the water pressure below the ice. It leads to the formation of floods on the ice surface during high tide. The ice surface becomes dry during low tide. Ice temperature and ice pressure on the joggle skirt are changed according to the semidiurnal cycle. Spectral analysis and correlation analysis are performed to analyse the loads caused by thermal expansion of the ice. A thermo-mechanical model of ice based on elastic-plastic rheology with thermal effect is used in numerical simulations of the observed phenomena. Numerical simulations are performed with finite element software.

This article is part of the theme issue ‘Modelling of sea-ice phenomena’.

## 1. Introduction

Thermal changes in ice cause its expansion or contraction and lead to loads on structures restricting ice deformations. Effects of thermal loads are mostly considered for the design of hydraulic structures in rivers, lakes and reservoirs [1]. Thermal ice pressure is one of the components of static ice load considered as a design parameter for hydropower dams (e.g. [2]). ISO 19906 [3] states that ‘based on full-scale measurements made in Russian and Canadian sea areas, sea ice does not expand appreciably for ice temperatures above −10°C for salinities greater than 3 ppt or above −7°C for salinities greater than 1 ppt.’ Indicative values of thermal stresses in the range of 150 to 300 kN m^−1^ can be used regardless of the ice thickness [4–6]. Thermal actions in freshwater ice are large in magnitude than those of sea ice.

The linear coefficient of thermal expansion (CTE) of freshwater ice 5.5 × 10^−5^ K^−1^ [7] shows that thermal pressure due to the expansion of confined ice with an elastic modulus of *n* GPa reaches 55*n* kPa when the ice temperature increases on 1°K. Processes of heat transfer and stress relaxation influence the significant reduction of this pressure if the temperature changes in the ice are caused by weather changes. In this case, the time for the increase of ice temperature at 1°K over entire ice thickness is big enough for significant relaxation of the stresses [8]. Therefore, thermal characteristics and creep rheology of ice should be taken into account for the calculation of thermal stresses [9].

Saline ice is a composite material including solid ice matrix containing liquid and gas inclusions. Liquid brine may exist inside sea ice in closed brine pockets and permeable channels [10,11]. Phase changes in closed brine pockets influence properties of sea ice thermal expansion. The effective coefficient of thermal expansion (ECTE) can be positive or negative depending on the temperature and amount of brine trapped in closed brine pockets [12,13]. In the case when all brine concludes in permeable channels the ECTE of sea ice is similar to the CTE of freshwater ice [14]. Since migration of brine through sea ice in the presence of temperature gradient influences the ice temperature then the thermal expansion of sea ice depends also on the ice permeability.

Migration of brine through the ice is caused by the pressure gradient. The permeability appears as a coefficient in Darcy's law describing the dependence of brine flow from the pressure gradient (e.g. [15]). The permeability of sea ice was studied for the description of gravity drainage processes and the input of brine and fresh water into the upper ocean in warm time of a year when the ice temperature is closed to the melting point [16–18]. Laboratory investigations of vertical permeability of saline ice in the ice tank performed by Marchenko *et al.* [19] show that cold ice with a surface temperature of −10°C is also permeable but its permeability is lower by 3–4 orders than in warm ice. Upward migration of the brine through the ice was caused by water overpressure in the tank when growing ice was frozen to the tank walls. Artificial pumping of the water pressure below the ice caused visible migration of brine through the ice and the increase of its surface temperature by 0.7°C [20].

In natural conditions, the formation of water overpressure below the ice can be caused by the action of tidal variations of the water pressure on confined ice with limited capacity to move in a vertical direction. Typical examples are related to ice foot and hinge zone near the shore line [21–24], partially grounded ice in very shallow regions [25] and ice confined in closed and relatively small areas (e.g. inside pool or cofferdam). In the present work, the processes in confined ice below the coal quay at Kapp Amsterdam (https://en.wikipedia.org/wiki/Cape_Amsterdam) are investigated and ice loads on the joggle skirts (JSs) of the quay are analysed. The paper is organized as follows. Physical processes in the confined ice and deformations of JSs are described in the second section of the paper. The third section is devoted to the description of the research site, installed scientific equipment and results of the field observations. Results of numerical simulations are performed in the fourth section. Results of modelling and field observations are compared and discussed in the last section.

## 2. Ice processes in the coal quay at Kapp Amsterdam

**Figure 1.**
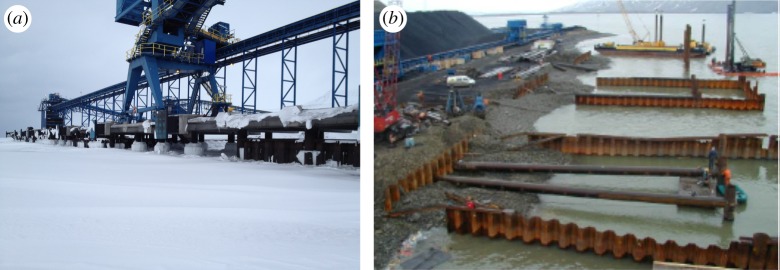
Coal quay in Kapp Amsterdam (photograph of the author) (*a*). Steel JSs connecting the piles of the quay during the reconstruction stage in 2000 (http://www.afgruppen.com) (*b*).

The coal quay at Kapp Amsterdam owned by Store Norske Spitsbergen Kullkompani (SNSK) was designed and reconstructed in 2000 by AF Anlegg Harbour for loading operations on vessels with dead weights up to 70 000 t. Coal boats provided the transport only during the ice-free season in the Van Mijen Fjord, and during the ice season the quay needed to survive without damage. The total length of the quay is 195 m; it is keyed in the seabed with vertical piles of 80 cm diameter and four steel JSs connecting some of the piles (figure 1). The piles are installed in two lines parallel to the shoreline and extending from each other by 8 m. Actual distances between neighbour piles in each line are 6 m. The soil was added at the bottom inside the skirts. Therefore, the sea bottom inside JSs is higher than sea bottom in the front of the quay by about 10 m. Sea water easily penetrates through JSs and the water levels inside and outside JSs are the same in the ice-free season.

**Figure 2.**
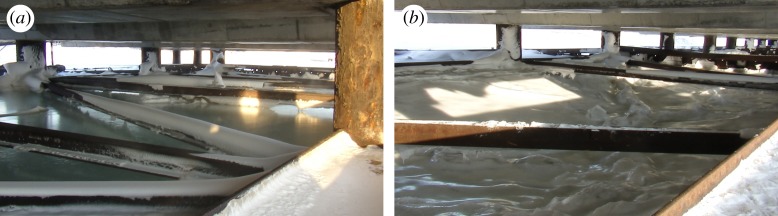
View on the ice surface inside JSs during high tide (*a*) and low tide (*b*). Photographs by the author.

**Figure 3.**
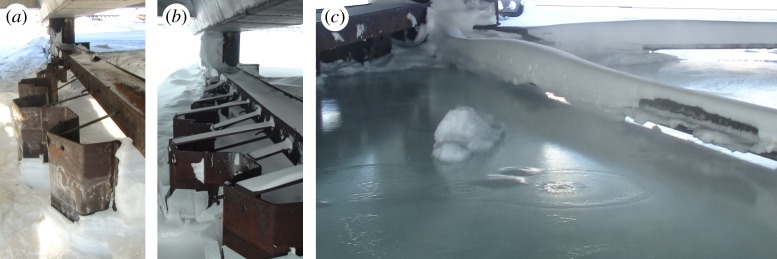
Displacements of the west wall of JSs in the alongshore direction in April 2008 (*a*) and March 2013 (*b*). Ring pointing out the location of sea water migration through the ice (*c*). Photographs by the author.

Sea ice in the fjord moves up and down together with sea water due to the semidiurnal tide. The ice inside JSs becomes frozen to the walls during the winter season and does not move like water. Synchronous measurements of the water pressure inside and outside JSs show similar semidiurnal changes of the pressure at the seabed [19]. Tide-induced overpressure at the ice bottom inside JSs influences floods on the ice surface during high tide when the tide amplitude is big enough (figure 2*a*). During low tide, the middle part of the ice inside JSs can be displaced downwards up to 0.6 m in comparison to its position during high tide (figure 2*b*). Displacements and deformations of JS sections perpendicular to the shoreline in the alongshore direction have been registered in different locations of the quay since 2008 [26]. The progressive deformations of the sections are prevented by the welding of steel holders between the skirt and the horizontal beams mounted to the vertical piles (figure 3*a*). Although the physical reasons for loads causing the JS deformations can be associated with both ice and soil actions the main focus of this study was on ice loads because of visual observations of ice deformations and floods inside JSs.

The ice thickness measured in different locations inside JSs was 2.2 m in April 2009 [19], 1.5 m in March 2013 [27] and 2.5 m in March 2015. The thickness of sea ice in the fjord near the quay was smaller by 0.9 m in these years. Large ice thickness inside JSs is explained by the water freezing at the ice surface during the floods. Locations of sea water migration through the ice inside JSs are visible by rings on the surface of flood water (figure 3*c*). Migration of sea water in the presence of vertical temperature gradient over the ice thickness gives a reason to assume that ice loads on JSs are caused by thermal expansion of the ice. At the same time, the ice inside JSs has visible vertical deformations which can lead to mechanical loads of the ice on JSs.

The hinge zone separating floating sea ice from the ice frozen to JS sections parallel to the shoreline is formed from the marine side and extended on approximately 5 m distance from JSs. Depending on the tidal amplitude, floods are observed also in the hinge zone during high tide. The surface of the hinge zone becomes dry during low tide. Usually, two cracks separating the hinge zone from sea ice and the ice frozen to JSs are clearly visible in the hinge zone. Ice blocks between the cracks are bent and rotated over the tidal cycle. In the ice-free season it was discovered that JS sections on the marine side of the quay are not mounted fast and move cyclically with the amplitude of a few centimetres perpendicular to their plane direction due to wave actions. Vertical piles supporting the quay are mounted fast and not moving.

## 3. Research site and results of observations

**Figure 4.**
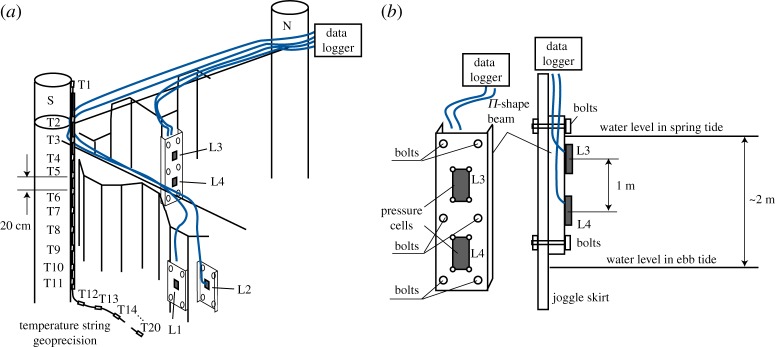
Scheme of the installation of Geokon Pressure Cells and Geoprecision temperature string in JSR (*a*). Locations of pressure cells L3 and L4 with respect to the water level (*b*). (Online version in colour.)

The research site was organized in the JS on the west part of the quay in the autumn of 2012. Further, this JS is named JSR. Geokon Pressure Cells (http://www.geokon.com/4850) LC1 and LC2 were screwed to two Π-shape beams, and each of the beams was mounted by four bolts on the JSR section parallel to the shore line from its marine and inner sides (figure 4*a*). Similar pressure cells LC3 and LC4 were screwed to the Π-shape beam and the beam was mounted on the section of JSR perpendicular to the shore line from its inner side (figure 4*a*). This section had the highest deformations, shown in figure 3*a,b*. LC1, LC2 and LC3 were mounted at the same distance from the water level, and LC4 was mounted 1 m deeper than LC1–LC3. It is assumed that LC1–LC3 measure pressure in the surface ice layer with a thickness of 0.5 m, and LC4 measures pressure in the middle layer of the ice (figure 4*b*). The works were performed during spring tide in November 2012. For the mounting of the Π-shape beams, JSR was drilled manually from a plastic rowing boat placed on the water below the quay. Cables from pressure cells LC1–LC4 were grouped together on the surface of the horizontal beams mounted to vertical piles and connected to the data logger fastened on the horizontal beam near the vertical pile N (figure 4*a*). Programming of the data logger and upload of the data were performed using LabView software with the laptop connected to the data logger.

Temperature string Geoprecision (http://www.geoprecision.com/en/produkte-en/temperaturmesskette-en) with 21 thermistors was placed inside a vertical steel pipe and the pipe was strapped to the vertical pile S in November 2012 (figure 4*a*). The first thermistor located inside the logger was extended 1 m from the next thermistor; 18 thermistors were distributed with 20 cm distance between neighbouring thermistors along the temperature string. The last two thermistors at the end of the temperature string extended from each other by 1 m. Vertical distances from thermistor 1 (TS1) and thermistor 2 (TS2) to the surface of ice near the pile S were around 1.5 and 0.5 m, respectively. Thermistors 1–4 (TS1–TS4) were above the ice surface. Thermistors 5–10 (TS5–TS14) were within the ice thickness when the ice inside JSR was thick enough (March–May). Thermistors 15–20 (TS11–TS20) were below the ice in the water and on the sea bottom. The temperature string supports wireless data transmission to the laptop via a USB dongle. Programming of the data collection was performed using GP5Shell software.

Registration of the tidal variations of sea level was performed with pressure and temperature autonomous recorders SBE 39 (https://www.seabird.com/moored/sbe-39plus-temperature-depth-recorder/family?productCategoryId=54627473774) placed on the sea bottom near the quay and below the quay inside JSR. Monitoring of floods on the ice surface inside JSR was performed with a Reconyx HC600 camera (www.reconyx.com) mounted on the vertical pile inside JSR. Four Geokon Pressure Cells (http://www.geokon.com/4800) were used in March 2015 for the monitoring of ice stresses in the central part of JSR synchronously with LC1–LC4.

**Figure 5.**
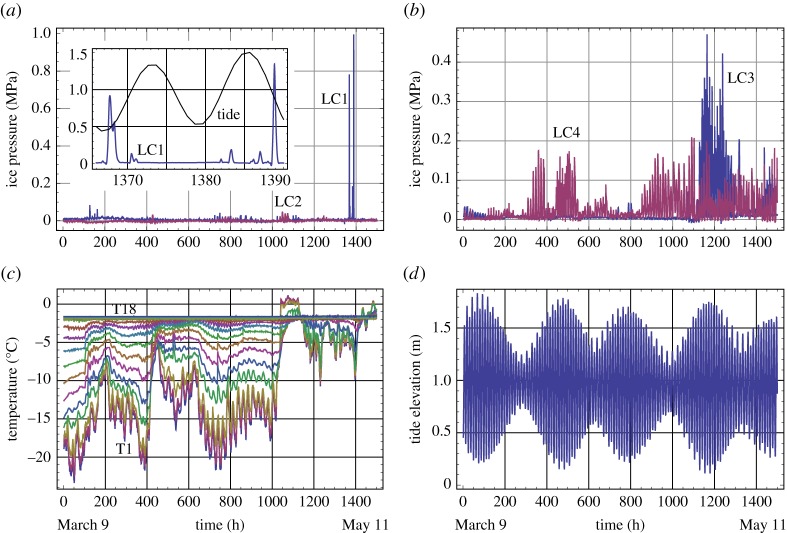
Plots of data recorded in winter 2013. Ice pressure versus the time recorded by L1 and L2 (*a*), L3 and L4 (*b*). Temperature records of the temperature string versus the time (*c*). Tidal elevation of sea level (*d*).

**Figure 6.**
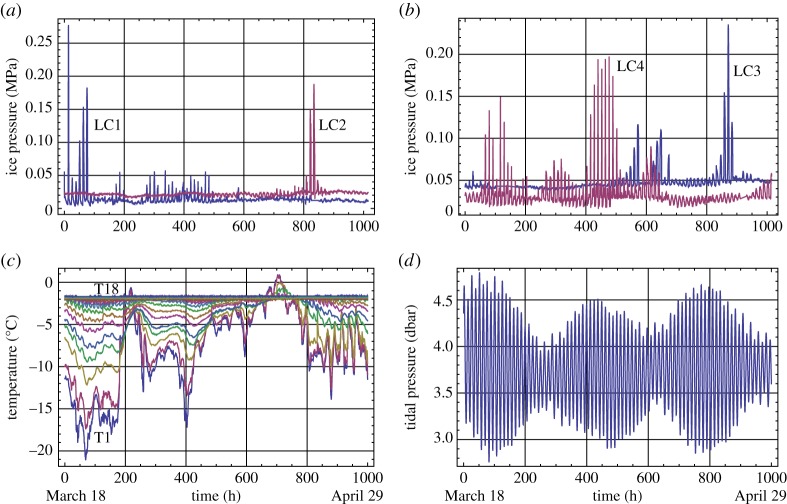
Plots of data recorded in winter 2015. Ice pressure versus the time recorded by L1 and L2 (*a*), L3 and L4 (*b*). Temperature records of the temperature string versus the time (*c*). Tidal pressure versus the time (*d*).

Figures 5 and 6 present data, acquired, respectively, during 2013 and 2015, on the ice pressure cells, temperature string and tidal measurements. During the warm winter of 2014, the ice did not freeze to JSR and moved up and down together with water as a set on unfrozen blocks. The ice pressure on JSR was not registered. Similar winters occurred in 2016 and 2017. Measurement of the water pressure at the seabed over the winter season was presented only in 2015. Tide-induced variations in the sea level in 2013 were taken from the tide table for Longyearbyen (http://www.kartverket.no/en/), and a 40 min phase shift was added to get the tide data for Svea Bay [28]. Sampling intervals of the measurements were 10 min for the ice pressure and water pressure and 30 min for the temperature. The tide tables provide information about the water level with a 1 h interval.

The highest ice pressures above 0.9 and 1.3 MPa were registered by LC1 on the marine side of JSR in two consequent tidal cycles at the beginning of May 2013 (inset in figure 5*a*). These ice pressure increases were of short duration, lasting about 2 h. They can relate to local effects of ice interaction with JSR in the hinge zone. Except for these two events, the ice pressures registered by LC1 and LC2 were much smaller than the ice pressures registered by LC3 and LC4. This can be explained by the damping of ice loads by the hinge zone and high compliance of JSR section on the marine side of the quay. The air temperatures in the beginning and middle of April were lower in 2013 than those in 2015 by almost 10°C. Therefore, sea ice was colder and stronger in 2013. Large ice pressures registered by LC3 and LC4 are attributed to the low temperature in 2013. At the same time, high ice pressures were registered in 2013 and 2015 when the air temperature was above −10°C. LC3 recorded ice pressures above 0.4 MPa at the end of April to the beginning of May 2013, and LC3 and LC4 recorded ice pressures above 0.15 MPa, respectively, at the end and at the beginning of April 2015.

**Figure 7.**
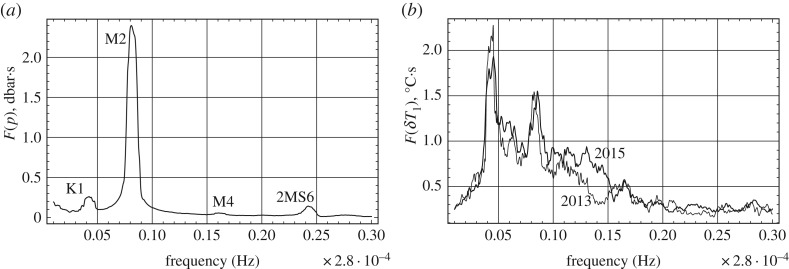
Spectra of tidal pressure in 2015 (*a*) and air temperature measured by TS1 in 2013 and 2015 (*b*).

Tidal changes of the water pressure below the ice and air temperature are the main factors influencing a change in the ice temperature and ice loads on JSR. The tidal signal has a sinusoidal shape corresponding to dominating semidiurnal tide M2 with a period of 12.42 h. Spectral analysis shows the existence of tidal constituents K1, M4 and 2M6 with periods of 24, 6.2 and 4.1 h (figure 7*a*). It corresponds well to the analysis of tidal constituents performed by Kowalik (private notes) based on the tide measurements in the Van Mijen Fjord from October 2007 to August 2009.

**Figure 8.**
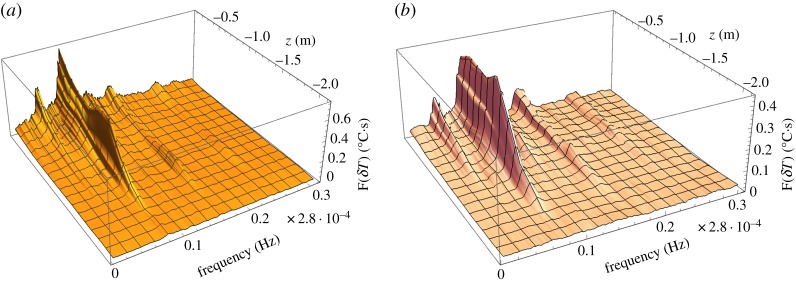
Spectra of the temperature recorded by TS4–TS14 in 2013 (*a*) and 2015 (*b*).

Spectral analysis was performed for the temperature fluctuations 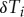 (*i *= 1–18) calculated with the formula
3.1
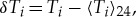

where 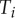 is the actual temperature measured by TS*i*, and 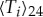 is TS*i*-temperature averaged over 24 h. Spectral analysis shows that fluctuations of the air temperature (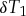) have spectral maxima at diurnal and semidiurnal frequencies (figure 7*b*). The highest spectral maximum at diurnal frequency corresponds to the air temperature variations caused by daily variations of sun radiation. The maximum at the semidiurnal frequency is explained by the influence of the floods on the ice surface on the air temperature below the quay. Spectral analysis of the temperature fluctuations recorded by TS4–TS6 shows the existence of spectral maxima at a diurnal frequency (figure 8). Spectral maxima at diurnal frequency are not visible on the records of TS7–TS14. This corresponds to the estimate of low bound of skin depth (18 cm) for air temperature signal at diurnal frequency [29]. The estimate does not include the influence of radiative heating. Figure 8 shows the existence of spectral maxima of the ice temperature fluctuations recorded by TS4–TS14 at the frequencies of tides M2, M4 and 2MS6. They are visible up to the depth of 1.7 m below the ice surface.

**Figure 9.**
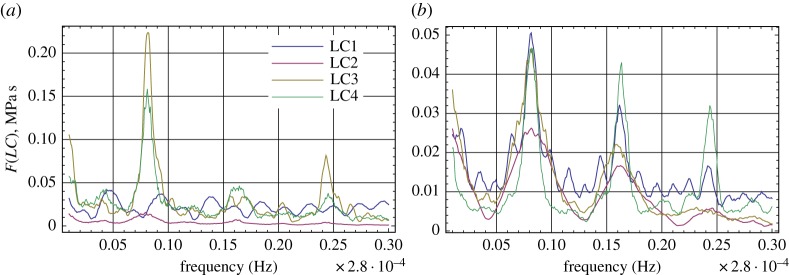
Spectra of ice pressures recorded by LC1–LC4 in 2013 (*a*) and 2015 (*b*).

Figure 9 shows spectra of the ice pressures recorded by LC1–LC4. In 2013 the records of LC3 and LC4 have spectral maxima at tidal frequencies M2, M4 and 2MS6, while the spectra of LC1 and LC2 do not have any maxima. In 2015, the records of LC1–LC4 have spectral maxima at the frequencies of M2 and M4, and only the record of LC4 has clearly visible spectral maxima at the frequency of 2MS6.

**Figure 10.**
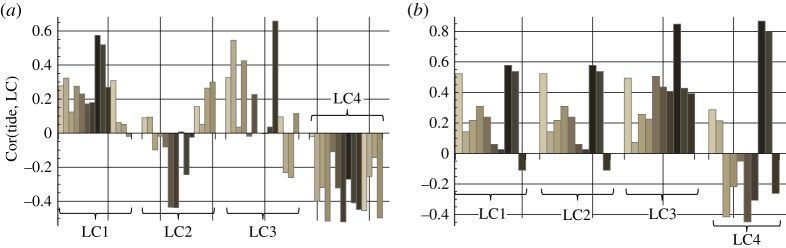
Correlations of ice pressures recorded by LC1–LC4 and tidal signals in 2013 (*a*) and 2015 (*b*).

Analysis of the correlations of the ice pressures recorded by LC1–LC4 with the records of tides is performed in figure 10. Measurement intervals in 2013 and 2015 were split into intervals of 100 h duration, and correlation analysis was performed for each of these intervals. Four columnar diagrams in figure 10 show the correlations of LC1–LC4 records with the records of tides. Each vertical column corresponds to the 100 h interval in the records. The number of columns calculated for each load cell (14—in 2013 and 10—in 2015) corresponds to the intervals of measurements (1400 h—in 2013 and 1000 h—in 2015). A positive correlation means that the ice pressure increases with the increase of the tidal pressure, a negative correlation means opposite time gradients of the ice pressure and the tidal pressure.

Since the ice pressures recorded by LC1 and LC2 were very small in 2013 (except two high pressure events measured by LC1) the information in figure 10*a* relates to the records of LC3 and LC4 showing mostly positive and negative correlations with the tide elevation of sea level. It means that LC3 registered the highest ice pressures during high tide, and LC4 registered highest ice pressures during low tide. There are 200 h of negative correlation of LC3 records with the records of tide elevation of sea level when high pressure amplitudes above 0.4 MPa were recorded at the end of April–the beginning of May 2013 (figure 5*b*). Figure 10*b* shows that in 2015 LC3 records have a positive correlation with the tide pressure. Records of LC4 showing relatively high pressure have a negative correlation with the tide pressure. In 2015 the records of LC1 and LC2 also show mostly positive correlation with the tide pressure.

**Figure 11.**
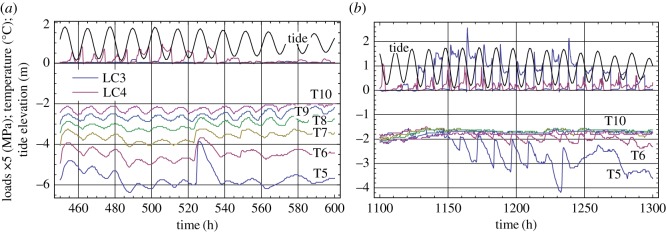
Ice pressure records by LC3 and LC4, ice temperature records by T5–T10, and tidal records on 27 March–3 April (*a*) and 23 April–2 May (*b*), 2013.

Figure 11 shows examples of records of LC3 and LC4 in 2013 with more details. Records of the temperature in the top ice layer of 1 m thickness and tide elevation of sea level are shown in the same figures. Local maxima of ice pressures recorded by LC3 correspond to high tide in figure 11*a* and located between high and low tide in figure 11*b*. Moreover, figure 11*b* shows that a significant part of ice pressures recorded by LC3 is spread around low tide. These events correspond to two columns showing a negative correlation of LC3 records with the tide records (figure 10*a*). Local maxima of ice pressures recorded by LC4 correspond to low tide in figure 11*a* and spread between low and high tides in figure 11*b*. Ice temperature records show local maxima of the temperatures during high tide and local minima during low tide in figure 11. Semidiurnal oscillations of the ice temperature recorded by TS5–TS10 are clearly visible in figure 11*a*. They are visible only on the records of TS5 and TS6 in figure 10*b* because of the higher air and ice temperatures (figure 5*c*). The ice temperatures at depths greater 0.5 m were almost at freezing point.

**Figure 12.**
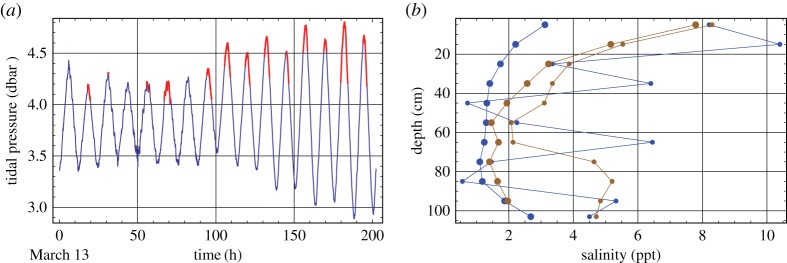
Flood intervals are marked by red on the record of tidal pressure versus the time in 2015 (*a*). Profiles of sea ice salinity measured in ice cores taken from two locations inside JSs during low (thick lines) and high (thin lines) tides in 2010 (*b*).

Figure 12*a* shows the flood times reconstructed by photographs taken with the Reconyx camera mounted inside JSR. Time lapse photographs were made with 15 min interval. The flood intervals are marked in red. The floods were observed during high tides. There is a tendency of flood duration increase with the increase of the tide amplitude. The depth of the flood water inside JSR was measured from 5 to 25 cm [19]. Ice cores where taken from the ice inside JSR during high and low tides and their salinity was measured. Measurements show a decrease of ice salinity during low tide and significant variations of the salinity in different locations of JSR (figure 12*b*). A decrease of ice salinity during low tide in comparison with high tide reaches several parts per thousand.

## 4. Numerical simulations

Thermo-mechanical ice loads caused by temperature changes in the ice are estimated using Bergdahl's rheological model [30]. The equation formulated by Cox [9] describes temporal evolution of thermal stresses in confined ice with zero strains at different depths. In dimensionless form it is written as follows:
4.1
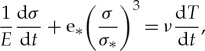

where 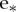 is the creep rate coefficient, 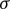
is reference creep stress, *E* is the elastic modulus of ice and 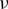 is the linear ECTE. The linear CTE of fresh ice is material constant equal to 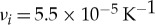 [7]. The absolute value of ECTE in negative range can be much greater than the value of CTE [12]. In the present study, ECTE is considered as a tuning parameter for the verification of numerical simulations according to the observations.

Numerical simulations were performed with finite element software (Comsol Multiphysics 5.2a). The program was prepared in a Structural Mechanics module using a model of linear elastic material with creep and thermal expansion implementing the model of Cox [9] for 3D simulations. The creep rheology is described by the Norton law with creep rate coefficient 10^−6^ s^−1^, reference creep stress 1 MPa and stress exponent 3. The elastic modulus and Poisson's ratio are, respectively, 2 GPa and 0.33. The computational domain is a rectangular block with sizes 20 m along the *x*-direction, 10 m along the *y*-direction and 2 m along the *z*-direction. The origin is located in the low corner of the computational domain, and the vertical coordinate *z* is directed upwards.

Fixed constrains are used as the boundary conditions at the lateral faces of the computational domain. The lateral faces correspond to the surfaces of ice in contact with JSs. The free surface boundary condition is used on the ice surface. The ice bottom is subjected by the action of hydrostatic pressure imitating tidal pressure
4.2
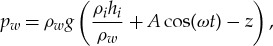

where water and ice densities are 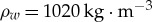, 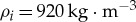, the tidal amplitude is 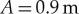 and 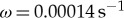 is the frequency of semidiurnal tide. The gravitational force on the ice is taken into account.

**Figure 13.**
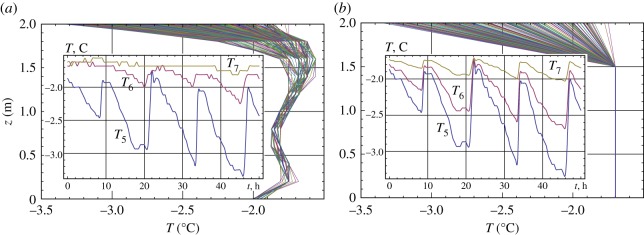
Vertical profiles of measured (*a*) and used in the simulations (*b*) ice temperatures. Insets show the time-history plots of three temperature profiles measured (*a*) and used in the simulations (*b*) during the specific time at the surface layer of the ice.

Thermal loading is specified according to the example shown in figure 11*b*. Numerical simulations were performed using the 60 h temperature records corresponding to the temperature records from 1150 to 1210 h in figure 11*b*. Temperature profiles measured by the temperature string in this time are shown in figure 13*a*. In numerical simulations, the temperature measured by TS5 specifies the surface temperature of the ice sheet. Temperatures measured by TS8–TS18 are almost constant. Therefore, thermal expansion is most significant in the surface ice layer of 0.5 m thickness. In numerical simulations, the ice temperature is assumed to equal to −1.7°C by 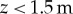, the linear temperature profiles are used when 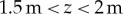 (figure 13*b*). The insets in figure 13 show measured and used in simulation dependencies of the ice temperatures from the time.

**Figure 14.**
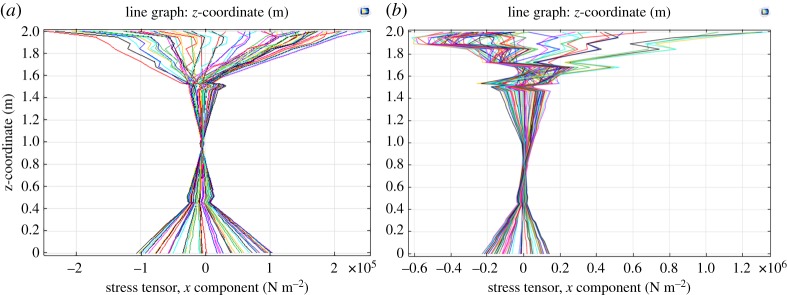
Vertical profiles of normal stress on the vertical segment: 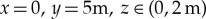
computed with 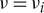 (*a*) and 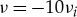 (*b*).

**Figure 15.**
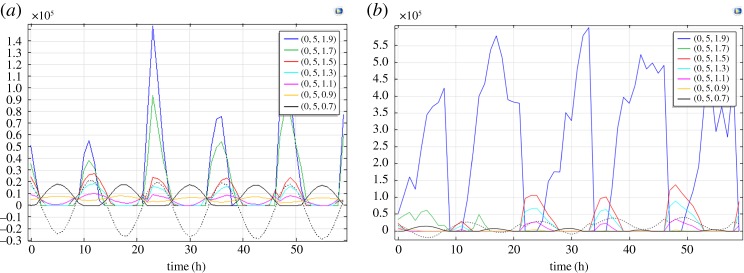
Results of numerical simulations performed with 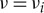 (*a*) and 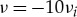 (*b*). Solid colour lines show the ice pressure (Pa) at the point with coordinates *x*, *y* and *z* shown in the legend. Black dotted lines show vertical displacement of the point with coordinates *x *= 10 m, *y *= 5 m and *z *= 2 m. The displacement (mm) is multiplied on 10^5^.

Figures 14 and 15 show results of numerical simulations. Figure 14 shows vertical profiles of normal stress on the vertical segment: 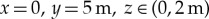. They are computed over each hour of the 60 h temperature records with 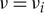 and 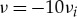. In figure 14*a* the stress range extends from −0.1 to 0.1 MPa in the bottom layer of ice and from −0.25 to 0.25 MPa in the surface layer of ice. In figure 14*b* the stress range extends from −0.2 to 0.2 MPa in the bottom layer of ice and from −0.6 to 1.35 MPa in the surface layer of ice. The difference of the stress ranges near the ice surface and ice bottom is explained by the influence of the thermal expansion. Computed tensile stresses are much higher than the tensile strength of sea ice (less than 0.2 MPa) measured in the full-scale tests near the coal quay [31]. Therefore, tensile cracks form near JSs in the surface ice layer, and the ice can be disconnected from JSs during low tide. The bottom ice layer is in compression near JSs during low tide.

Figure 15 shows ice pressures computed in different points of the vertical segment versus the time. The point coordinates (*x*,*y*,*z*) are shown in the legends. According to the definition the ice pressure equals 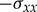 when 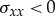 (compression) and 0 when 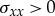 (tension). Black dotted lines show vertical displacement of the ice in the middle of the computational domain at *x *= 10 m and *y *= 5 m. In figure 15*a* the ice pressure phases follow the phase of the vertical displacement. Therefore, positive thermal expansion (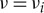) does not explain the increase of the ice pressure during low tide shown in figure 11*b*. In the case of negative thermal expansion (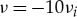) the ice pressure computed in the points with coordinates *z *= 1.9 m and *z *= 1.7 m has opposite phase to the tide, i.e. pressure maxima are realized during low tide when the vertical displacements of the ice are negative (figure 15*b*). Thus, negative thermal expansion in the surface ice layer explains the formation of ice pressure on JSs shown in figure 11*b*.

Amplitudes of computed vertical displacements of the ice are below 0.5 mm. This is much smaller than the observed values up to 0.6 m. The large displacements are related to the formation of ice failure zones in the ice. The model should be modified to take into account ice failure effects. Another correction of the model is related to the more accurate formulation of the creep rheology of sea ice taking into account its granular structure [32].

## 5. Discussion and conclusion

Ice pressures on JSR in the coal quay in Spitsbergen and ice temperatures were recorded during two winter seasons in 2013 and 2015. The ice loads are created by confined ice sheet trapped inside JSR. The ice thickness was about 2 m. Three pressure cells recorded the ice pressure on JSR in the surface ice layer of 0.5 m thickness, and one pressure cell recorded the ice pressure on JSR 1 m deeper than three other cells. Two ice pressure increases above 0.9 and 1.3 MPa were registered in the surface ice layer on the marine side of JSR at the beginning of May 2013. Except for these two events with less than 2 h duration the recorded ice pressures oscillate with semidiurnal frequency. The amplitudes of the pressure oscillations were below 0.2 MPa in most of the records except the record of approximately 200 h duration at the end of April to the beginning of May 2013 when the pressure amplitude exceeded 0.4 MPa in the surface ice layer. Spectra of the pressure records correspond to tidal constituents M2, M4 and 2M6 with periods of 12.42, 6.2 and 4.1 h. Correlation analysis shows that highest pressures in the surface ice layer are realized during high tide except for the 200 h record in 2013 when the highest pressures in the surface ice layer were recorded during low tide.

Spectra of temperature fluctuations have maxima corresponding to the same tidal constituents visible up to a depth of 1.7 m below the ice surface. The temperature fluctuations are explained by sea water migration through the ice under the action of tidal pressure on the ice bottom. Spectral maxima of the ice temperature at diurnal frequency are visible in the surface ice layer up to a thickness of 0.5 m. They are related to diurnal variations of the air temperature and sun radiation. Correlation analysis shows that the ice temperature increases when the water level in the sea increases and decreases when the water level decreases. Migration of sea water through the ice influences floods on the ice surface during high tide when the tide amplitude is big enough. The flood durations increase with the increase of the tide amplitude. The ice surface becomes dry during low tide. The ice salinity during low tide is smaller than the ice salinity during high tide by several parts per thousand.

Observations show that the ice inside JSR bends downwards during low tide and the ice displacement in the middle of JSR reached 0.6 m. This influences the pressure of the bottom ice layer on JSR during low tide, and the surface ice layer during high tide. Similar loads are caused by thermal expansion of the ice in the case of the positive CTE. Numerical simulations with the elastic-plastic model of ice taking into account thermal expansion are performed when the ice temperature changes according to the 60 hour temperature records during April 2013. Amplitudes of ice pressure on JSs are estimated below 0.25 MPa when ECTE of sea ice equals to CTE of fresh ice. The increase of ice pressure amplitudes up to 0.6 MPa during low tide was reproduced by numerical simulations with the elastic-plastic model of ice where ECTE of sea ice was negative in the surface ice layer, and its absolute value was 10 times greater than the CTE of fresh ice.

Negative thermal expansion of saline ice with salinities of 6 and 9.4 ppt was reproduced in laboratory experiments when the ice temperature was higher than −5°C [13,20]. The values of ECTE were found to be about −10^−4^ K^−1^. Recent *in situ* investigations of the influence of ice compression on ice permeability performed on sea ice near the coal quay demonstrated strong reduction of the permeability in compressed ice [33]. Based on this study the origin of high ice pressures on JSR during low tide can be explained as follows. The surface ice layer inside JSR is compressed during low tide and its salinity is relatively high (e.g. 8 ppt in figure 12*b*). The compression influences a reduction of ice permeability and a shift of ECTE to negative values. Negative thermal expansion of the surface ice layer influences high ice pressure on JSR during low tide.

## Data accessibility

This article has no additional data.
